# Development of Flexible Ion-Selective Electrodes for Saliva Sodium Detection

**DOI:** 10.3390/s21051642

**Published:** 2021-02-26

**Authors:** Hyo-Ryoung Lim, Soon Min Lee, Musa Mahmood, Shinjae Kwon, Yun-Soung Kim, Yongkuk Lee, Woon-Hong Yeo

**Affiliations:** 1 George W. Woodruff School of Mechanical Engineering, Center for Human-Centric Interfaces and Engineering, Institute for Electronics and Nanotechnology, Georgia Institute of Technology, Atlanta, GA 30332, USA; hlim308@gatech.edu (H.-R.L.); musamahmood@gatech.edu (M.M.); skwon64@gatech.edu (S.K.); ysk@me.gatech.edu (Y.-S.K.); 2 Department of Pediatrics, Gangnam Severance Hospital, Yonsei University College of Medicine, Seoul 06273, Korea; smlee@yuhs.ac; 3 Department of Biomedical Engineering, Wichita State University, Wichita, KS 67260, USA; yongkuk.lee@wichita.edu; 4 Wallace H. Coulter Department of Biomedical Engineering, Georgia Institute of Technology and Emory University School of Medicine, Atlanta, GA 30322, USA; 5 Parker H. Petit Institute for Bioengineering and Biosciences, Institute for Materials, Neural Engineering Center, Institute for Robotics and Intelligent Machines, Georgia Institute of Technology, Atlanta, GA 30332, USA

**Keywords:** saliva-to-blood sodium levels, electrochemical sensor, ion-selective electrode, non-invasive diagnosis

## Abstract

Saliva can be used for health monitoring with non-invasive wearable systems. Such devices, including electrochemical sensors, may provide a safe, fast, and cost-efficient way of detecting target ions. Although salivary ions are known to reflect those in blood, no available clinical device can detect essential ions directly from saliva. Here, we introduce an all-solid-state, flexible film sensor that allows highly accurate detection of sodium levels in saliva, comparable to those in blood. The wireless film sensor system can successfully measure sodium ions from a small volume of infants’ saliva (<400 µL), demonstrating its potential as a continuous health monitor. This study includes the structural characterization and error analysis of a carbon/elastomer-based ion-selective electrode and a reference electrode to confirm the signal reliability. The sensor, composed of a pair of the electrodes, shows good sensitivity (58.9 mV/decade) and selectivity (log K = −2.68 for potassium), along with a broad detection range of 5 × 10^−5^ ≈ 1 M with a low detection limit of 4.27 × 10^−5^ M. The simultaneous comparison between the film sensor and a commercial electrochemical sensor demonstrates the accuracy of the flexible sensor and a positive correlation in saliva-to-blood sodium levels. Collectively, the presented study shows the potential of the wireless ion-selective sensor system for a non-invasive, early disease diagnosis with saliva.

## 1. Introduction 

The future of non-invasive prognosis using salivary ions hinges on achieving clinical accuracy, patient comfort, and easy access to monitoring data. Wireless, wearable sensor systems can satisfy the requirements by offering non-invasive, real-time, continuous monitoring of target ions and signals [1,2,3]. A recent study introduced a fully wireless, portable sensor system detecting salivary sodium ions, enabled by biocompatible materials, miniaturized electronics, and thin-film electrochemical ion sensors [4,5]. All-solid-state ion-selective electrodes (SS-ISEs), the most well-known candidates for a wearable potentiometric sensor, play a significant role in achieving a small areal of footprint per device without bulky detector systems. Nonetheless, despite the enormous potential improvements from a technical perspective, the small SS-ISE device has yet to receive proper attention in diagnostic capabilities.

There are many examples of detecting salivary protein biomarkers in a qualitative way [6]. While some biomolecules require a complicated lab analysis, the ion-level measurement via potentiometric mechanism is relatively straightforward. Given that the potentiometric sensing principle is based on the transport of ions from high to low concentrations on the surface that creates a potential difference [7], the whole system only needs a pair of an ISE and RE along with a voltage reading device. However, the standard detection methods of ion levels still rely on bulky devices. One such example is an electrochemical sensor whose functionality is achieved by a predetermined stabilization of well-defined rigid surfaces, involving a protective housing and/or glassy bulb. Therefore, the system often requires a large volume of sample (up to 100 mL) to soak the sensors’ surface [8]. In addition, the commercial sensors need a discrete data processing unit and cables to calibrate the sensor and measure the ion levels, making the actual device cumbersome.

Despite the poor accessibility associated with the bulky sensor system, some clinical studies have demonstrated the usefulness of quantitative detection of salivary ion levels. While the blood sodium levels (135–145 mM/L) are known for reflecting blood pressure and heart failure, often predicting morbidity and mortality [9], the salivary sodium ion levels (4 to 37 mM/L) have been measured in clinical settings [10,11,12,13]. Some studies showed a positive correlation in blood-to-saliva sodium ion levels via flame photodetector [14], radiometer [12,15], and photometer [16], paving the way for the non-invasive diagnosis of cystic fibrosis, diabetes, and cardiovascular diseases. However, such optical sensors require a rigid, bulky detector that may involve other specialists and separate devices, unlike SS-ISEs [17]. 

The advancement in SS-ISEs has successfully removed the use of the fragile parts and minimized the sensor’s size by direct contact between all-solid-state layers of an electron collector, ion-to-electron transducer, and ion-selective membrane [7]. By developing a materials’ structure and detection system, the SS-ISE functioned well through a few steps of pre-stabilization for the surface hydration, calibration in the range of ion levels of interest, and detection of the analytes via a small voltage-reading circuit [18]. Indeed, the urgent need is to compare the ion levels in blood with the non-invasive salivary ions measured from a small SS-ISE system in clinical settings [19]. Aside from a lack of experimental evidence from the SS-ISEs, no previous studies have assessed the difference in the sodium ion levels in premature infants’ blood and saliva. For the most precise comparison, an appropriate sampling protocol is necessary, along with an accurate, user-friendly system. Although often ignored, salivary biomarkers’ diagnostic accuracy highly depends on patient and environmental factors, the type of glands, and sampling methods [20,21,22]. The difference in sodium-ion levels between whole and accumulated saliva reaches tens of mM/L [23], which may deter the prognosis, diagnosis, and treatment.

Here, we compare the sodium levels in saliva obtained from a small electrochemical film sensor with serum analyzed in a commercial sensor. Our film sensor functions with a small volume of infants’ saliva (<400 μL), demonstrating its potential as a non-invasive tool, even when involving subjects unable to collect their saliva. We conduct structural characterization and error analysis for our previously developed carbon/Ecoflex ISE and a reference electrode to confirm their signal reliability. In addition, our wireless data acquisition unit adopts a low-energy Bluetooth module and an operational amplifier (OpAmp), which is suitable for long-term monitoring in wearables. Unstimulated whole saliva was collected from infants while using a catheter into the mouth with the same amount of water. The comparison results indicate a positive correlation in saliva-to-blood sodium levels as well as in film-to-commercial electrochemical sensors. This study can collectively provide evidence for the non-invasive, easily assessable infant saliva diagnosis with film electrochemical systems.

## 2. Materials and Methods

### 2.1. Materials

First, 4-tert-butylcalix[4]arene-tetraacetic acid tetraethyl ester (sodium ionophore X), bis(2-ethylhexyl) sebacate (DOS), poly(vinyl chloride) (PVC), tetrahydrofuran (THF), hydrochloric acid (HCl), silver nitrate (AgNO_3_), and polyvinyl butyral (PVB) were purchased from Sigma Aldrich. Sodium tetrakis-[3,5-bis(trifluoromethyl)phenyl] borate (NaTFPB) was purchased from Alfa Aesar. Sodium chloride (NaCl), potassium chloride (KCl), calcium chloride dihydrate (CaCl_2_·2H_2_O), and magnesium chloride hexahydrate (MgCl_2_·6H_2_O) were purchased from Fisher Chemical. Carbon black (Vulcan XC 72R) was obtained from FuelCellStore. Silicone elastomers, including Ecoflex 00-30 and polydimethylsiloxane (PDMS, Sylgard 184), were obtained from Smooth-On and Dow Corning, respectively. Fast-drying silver paint was purchased from Ted Pella. 1X Phosphate-buffered saline (PBS; pH = 7.4) was obtained from Gibco by Life Technologies (Carlsbad, CA, USA).

### 2.2. Electrodes Fabrication

A film ISE, composed of an Au electron conductor, carbon/Ecoflex transducer, and sodium ion-selective membrane (ISM), was formed on a flexible Au electrode. The Au electrode was fabricated by a conventional microfabrication method (Figure S1). The fabrication of the carbon/Ecoflex transducer was developed in our prior work [5]. Briefly, we formed the carbon/Ecoflex by mixing 9 wt % carbon and 91 wt % Ecoflex in isopropyl alcohol (IPA), coating the paste on the Au, and curing at 150 °C for 1 h. The sodium-ISM cocktail was prepared by mixing sodium ionophore X (2.67 mg, 1 wt %), DOS (174.53 mg, 33 wt %), PVC (88 mg, 65.45 wt %), NaTFPB (1.47 mg, 0.55 wt %) in 2 mL of THF. The weight ratios of the membrane ingredients were fixed at 1:33:65.45:0.55 [24,25]. The mixtures were vortexed to make a homogeneous solution. The carbon/Ecoflex electrode was coated with a 3 μL of the membrane cocktail and dried overnight in the air.

A film reference electrode (RE) was formed on a Si wafer. A 500-nm Ag/50-nm Ti was e-beam evaporated and vacuum annealed at 320 °C for 30 min. The Ag surface was chlorinated by cyclic voltammetry from 0.1 to 0.3 V [5]. A carbon counter electrode (CE) and a commercial glass-type RE were used for the chlorination setup via a Gamry potentiostat (Interface 1010E, Gamry Instruments Inc., Warminster, PA, USA). The Ag/AgCl film was coated with a membrane mixture composed of 78.1 mg PVB, 50 mg KCl, 50 mg AgNO_3_, and 1 mL methanol [26]. After curing the surface with ultra-violet radiation (395–405 nm), the surface was coated by 2 μL Nafion to prevent leakage of salts. The resulting RE was dried at room temperature overnight.

### 2.3. Small Sensor System

A sensor dish was prepared by pouring the mixture of 1:1 wt % Ecoflex 00-30 into a polystyrene dish. After curing the elastomer for 30 min at 70 °C, the ISE and RE were deployed on the surface. A commercial sodium ISE (CNT_ISE M023, NT Sensors, El Catllar, Spain) and RE (NT MRX11, NT Sensors) were fixed next to the film sensors with Ecoflex for comparing the results of ion levels. Each electrode was connected to a jumper wire with an Ag paint. The connection was dried in air for 30 min and covered with 1:10 wt % PDMS. The encapsulation polymer was cured at room temperature overnight. We formed Ecoflex wells around the sensors to load the saliva samples on the sensor surface.

The sensors were connected to a voltage-reading circuit with jumper wires. The circuit was designed for measuring voltage signals from a potentiometric sensor and wireless transmission of sensing data. While the circuit design followed our prior work [5], the power consumption of a Bluetooth low-energy chip (nRF52832) and amplifier (OpAmp) was further investigated by measuring voltage and current after removing each component from the circuit via a multimeter (details of the circuit design and list of components in Figure S2 and Table S1). Battery life was obtained by recording data during wireless transmission using a rechargeable lithium-ion battery (3.7 V, 400 mAh, Digi-Key, Theif River Falls, MN, USA).

### 2.4. Characterization of Sensor

Scanning electron microscope (SEM) images for the ISE and RE were taken using equipment (Hitachi 8230, Tokyo, Japan). Sodium chloride solutions with different concentrations were prepared for calibration of the sensor (10^−3^ to 1 M). The range covers an average level in human saliva (4 to 37 mM). Long-term stability and conductivity study were conducted with a potentiostat. The interference study against potassium, magnesium, and calcium ions used the sensor voltages measured in 0.1 M chloride solutions for 10 min. A commercial solid-state RE (NT MRX11, NT Sensors) was used as CE/RE to obtain the voltage stability of the film RE in the different sodium chloride solutions.

### 2.5. Sampling of Blood and Saliva

The present study used saliva samples from sixteen infants in the neonatal intensive care unit of Gangnam severance hospital at the Yonsei university college of medicine (IRB# 3-2019-0206). The infants showed a median of 28 weeks of gestation (range 24–38 weeks) and a median of 1.1 kg of birth weight (range: 0.6–3.2 kg). Unstimulated whole saliva was collected by a saliva suction catheter using 4 mL of deionized (DI) water. We compared sodium ion levels measured from our film sensors with blood ion levels measured with a Abbott i-STAT clinical chemistry analyzer (Abbott, Abbott Park, IL, USA). The blood and saliva ion levels were measured within an hour apart on the same day. The ion level data were expressed in milliequivalents per liter (mEq/L) and evaluated using Spearman’s correlation coefficient analysis. By definition, 1 mEq is equal to 1 mM in the case of the monovalent sodium ion [27].

### 2.6. Measurement of Ion Levels

The surface of the sensor was conditioned overnight in a sodium chloride solution (concentration: 10^−3^ M) before measurements. The sensor was cleaned with DI water and calibrated using different sodium chloride solutions (10^−3^, 10^−2^, and 10^−1^ M) for 5 min each. The calibration was conducted every time right before measurement for salivary ion levels. We performed at least three times cleaning after saliva tests to remove any residues from the surface. For comparison between our film and the commercial ion sensors, the same saliva samples were dropped on the two sensors’ surfaces and analyzed subsequently using the same calibration and measurement protocol.

## 3. Results and Discussion

### 3.1. Sodium Ion Sensor System for Saliva Testing

**Figure 1:**
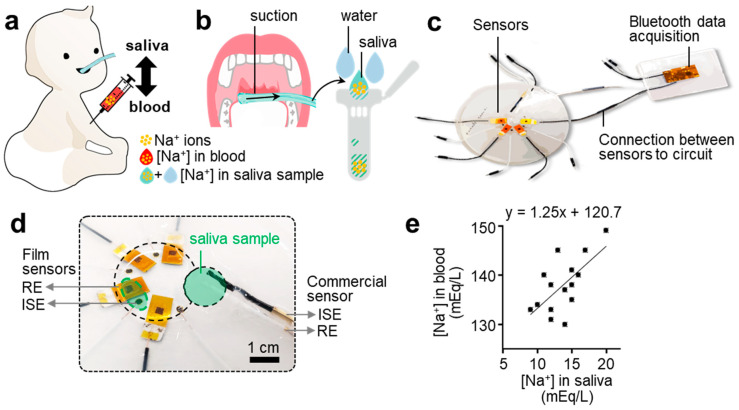
Flexible electrochemical sensor system for non-invasive detection of salivary sodium ions. (**a**) Schematic illustration of an infant subject to extract both saliva and blood samples to validate the sodium sensor performance. Direct, invasive blood sampling is less favored due to discomfort, bruising, and infection. (**b**) Illustration of a non-invasive saliva sample collection process. (**c**) Photo of an integrated system, including thin-film electrochemical sensors and a low-power wireless circuit for data acquisition. (**d**) Zoomed-in view of a salivary sample loading area with both film sensors and a commercial sensor, composed of a pair of an ion-selective electrode (ISE) and reference electrode (RE), for the performance validation. The required sample volume for the thin-film sensor system is less than 400 μL, which is much smaller than the requirement (over 1 mL) of a commercial device. (**e**) Positive correlation results of sodium ion levels between blood samples and saliva samples with the flexible sensor for sodium detection (*n* = 14).

Figure 1 shows an overview of non-invasive saliva monitoring using our small electrochemical sensor system. One of the most prominent saliva testing features is non-invasive sampling, distinguishing the saliva from the blood (Figure 1a). As illustrated, this approach protects infant subjects from transient discomfort, infection, and bruising. To collect saliva from the infant subjects who cannot spit it into a container, we inserted a tube in the oral cavity and suctioned unstimulated whole saliva (Figure 1b). As depicted in the schematic, this method involved water use to help collect the highly viscous saliva. Although there is no standardized method, the sampling of unstimulated whole saliva is known as the best to minimize the dilution of analytes in saliva [22,28,29]. The concentration ratio of saliva and water was kept constant by controlling a flow rate.

We compared the sodium ion levels in the collected saliva sample measured from our sensor with those of blood. Figure 1c shows the sensor device, composed of a sensor dish and wireless data acquisition unit fixed on separate dishes and connected with jumper wires. The circuit includes a Bluetooth low-energy module and 2.45 GHz chip antenna circuit, enabling a long-range wireless data transmission. The small form factor of the electronic system offers a portable detection of saliva, unlike the conventional table-top optical devices. In Figure 1d, a photo shows an enlarged view of the sensor part. The mobile sensor dish (15 cm in diameter) includes four film-type ISEs, REs, and a commercial ISE for the saliva testing and evaluation of film sensor readings. Wire connections except for those in the measurement area are encapsulated with an elastomer to eliminate leakage error. The resulting device shows the difference in the amount of sample required to measure the ion concentration in the film and commercial sensor. Figure 1e shows the result of a positive correlation in blood-to-saliva ion levels measured from multiple subjects. Such biological fluids are described in mEq/L, as in medical tests involving small amounts of analytes. In the monovalent sodium ion case, the mEq/L and mM are interchangeable by definition [27]. Our film sensors can accurately measure sodium ions from a sample volume as small as 400 μL, which is lower than that required to soak the rod-type commercial sensors.

### 3.2. Film Sensor Structure and Sensing Capability

**Figure 2:**
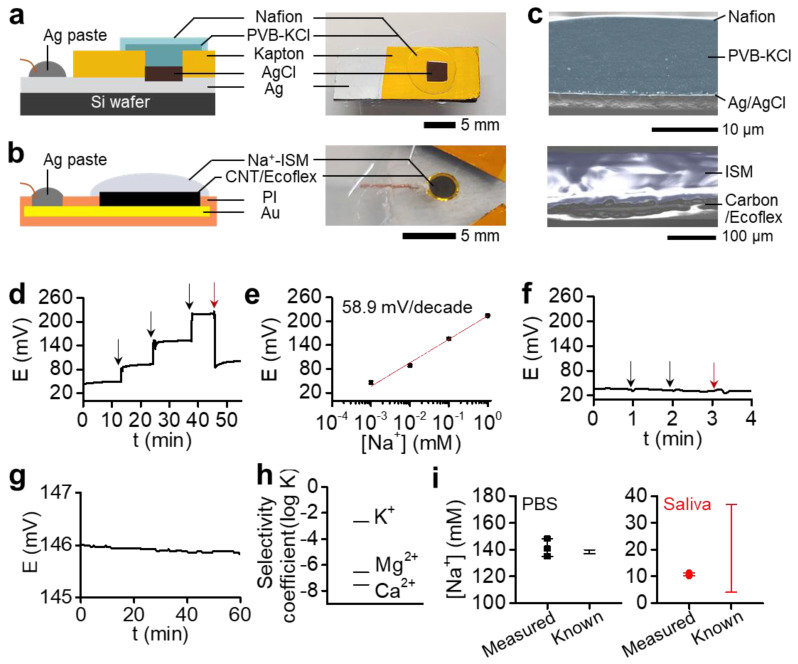
Characterization of a low-profile reference electrode (RE) and ion-selective electrode (ISE). (**a**,**b**) Schematic illustrations and photos of multilayered structures of (**a**) an Ag/AgCl/PVB-KCl/Nafion RE and (**b**) a carbon/Ecoflex-based ISE. (**c**) Cross-sectional SEM images of the RE (left) and ISE (right) in (**a**,**b**), respectively. (**d**) Voltage transient measured from an ISE in 10^−3^–1 M sodium chloride solutions (black arrows) and saliva (red). (**e**) Linear correlation between measured voltages and sodium levels, showing a sensitivity of 58.9 mV/decade. (**f**) Voltage stability of a RE continuously measured with 10^−3^ and 10^−2^ sodium chloride solutions (black arrows) and saliva (red). The RE was used as a working electrode (WE) with a commercial RE as a RE/carbon counter electrode (CE) in a two-electrode system. (**g**) Voltage stability of a sensor composed of a pair of SS-ISE and RE in 0.1 M sodium chloride (0.2 mV/h). (**h**) Selectivity coefficient (log K) measured for three interference ions that are abundant in saliva. (**i**) Detection of sodium ion levels by using the flexible sensor system in phosphate-buffered saline (PBS) and saliva (*n* = 3) and comparison of the measured data to known values.

Figure 2 shows the structural characteristics and sensing capabilities of our film sensor composed of a pair of RE and ISE. Figure 2a shows a film RE including multiple layers of Ag/AgCl coated with PVB-KCl/Nafion. We used the same structure in our previous study on the flexible circuit [5]. However, considering that the flexible RE often contributes to an unstable signal in a potentiometric detection setting [30], the device’s sensing reliability was further improved by making a rigid film on top of the Si wafer. The rigid RE electrode was prepared by an e-beam evaporation of Ag, vacuum annealing, surface chlorination, and membrane coatings (details in Materials and Methods). Except for the electrode (<5 mm) and wire contact area, the surface was insulated with Kapton tape due to its excellent chemical stability. Figure 2b captures a film ISE structure using a CNT/Ecoflex transducer that shows good sensitivity and voltage stability (see below). Figure 2c provides an SEM photograph of the cross-sectional microstructure of the RE and ISE, where the membrane structure is highlighted. The RE morphology (left) clearly captures the thick layer of a salt bridge, PVB-KCl, covered by Nafion. The image for the ISE structure (right) shows a 100-μm-thick ISM covering the bottom transducer layer without delamination due to the hydrophobicity of the carbon/Ecoflex.

Figure 2d,e present a voltage transient and linearity from the fabricated ISE. The real-time voltage data were measured for 10 min in different sodium chloride solutions (10^−3^ to 1 mM) and the saliva. The sensitivity obtained through calibration is 58.9 mV/decade near a theoretical (Nernstian) sensitivity (*n* = 3) (Figure S3a). While the repeatability was confirmed in the range covering the salivary sodium ion levels, the detection range and limit were determined to be 5 × 10^−5^ ~ 1 M and 4.27 × 10^−5^ M, respectively (Figure S3b). The sensor characteristics contribute to the voltage stability of the RE. As shown in Figure 2f, RE showed stable voltage values in the sodium chloride solutions of 10^−3^, 10^−2^, and saliva. The changes in voltage are negligible compared to those observed in the ISEs. As a result, the ion sensor composed of a pair of SS-ISE and RE showed good voltage stability and ion selectivity. Figure 2g captures the sensor’s stable voltage measured in a 0.1 M sodium chloride solution for an hour, showing the minimal voltage change by 0.2 mV/h. Selectivity analysis, carried out with the most abundant interference ions in body fluids, resulted in good coefficient (log K) values of −2.68, −7.52, and −6.57 for K^+^, Mg^2+^, and Ca^2+^, respectively (Figure 2h). The selectivity coefficient is calculated by a separate solution method by using the potential at 0.1 M [31,32], while detailed calculation methods are provided in our previous works [18,33]. Collectively, the sensing capability and signal stability are achieved by constructing multiple layers of the electrode structure, improving interlayers’ adhesion and encapsulating all contact areas that can cause signal noises. Using the sensor system, we could successfully measure the sodium ion levels in PBS and saliva samples (Figure 2i). The resulting levels from three tests show calculated values of 141 ± 7 mM and 11 ± 1 mM in PBS and saliva, respectively. The sodium levels of PBS show only <4 mM difference to known values, confirming the acceptable upper limit for the difference between the two methods [34]. The measured saliva levels of 11 mM are in the reported range of the sodium levels in saliva [22]. Future work regarding sensor fabrication will adopt a printing of the flexible ion-to-electron transducer for large-scale production and great reproducibility.

### 3.3. Low-Power Circuit for Wireless Continuous Data Transmission

**Figure 3:**
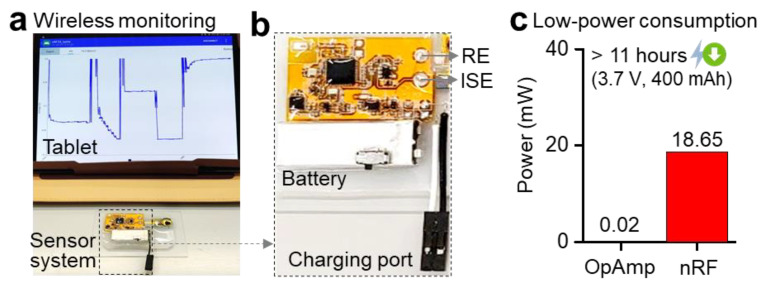
Wireless, low-power sensor system. (**a**) Photo of a wireless sensor system and a portable tablet, using Bluetooth for data acquisition. The tablet shows real-time voltage changes from sodium ions, measured by the sensor system. (**b**) An enlarged photo showing the sensor system, including multiple chip components on a flexible circuit, sensor connection pads for RE and ISE, and a rechargeable battery with a switch. (**c**) Power consumption measured by two main chips (OpAmp and nRF microcontroller) for wireless voltage monitoring. The device operation time with continuous detection of sodium ions is over 11 h with a rechargeable battery.

Figure 3 shows a flexible, low-power potentiometric sensor system using a Bluetooth-low-energy module and OpAmp. As shown in Figure 3a, the device was connected to a single sensor for real-time calibration and measurement capability via a portable tablet. When used with a single sensor and a rechargeable battery with a switch, the total device occupies an area of 4 × 5.5 cm^2^. This small footprint makes it easy for caretakers or patients to test themselves and providers to increase the number of sensors. Considering that the electrochemical sensors are cost-efficient yet have a relatively short period of a lifetime (in our case, three weeks), such a system will help users replace the sensors without management.

A photo in Figure 3b captures an enlarged view of the device, showing a Bluetooth module (nRF-52832) for wireless transmission, an OpAmp module measuring the sensor’s voltage, and a rechargeable battery along with a charging port. The ISEs are connected directly or via jumper wires to improve the device’s flexibility and mobility [35,36]. To verify the device’s low power consumption, we measured the output voltage and current while removing the Bluetooth chip and amplifier from the circuit. Calculated power consumptions from each component are 18.65 and 0.02 mW for nRF and OpAmp, which is directly related to battery life (Figure 3c). When connected with a sensor during wireless transmission, the device shows a wireless, continuous recording of sodium ions over 11 h using a rechargeable lithium-ion battery (3.7 V, 400 mAh). While the device operation time depends on the energy capacity, the miniaturized electronic system can be readily integrated with other types of portable or wearable sensor devices.

### 3.4. Correlation between Blood and Saliva Ion Levels

**Figure 4:**
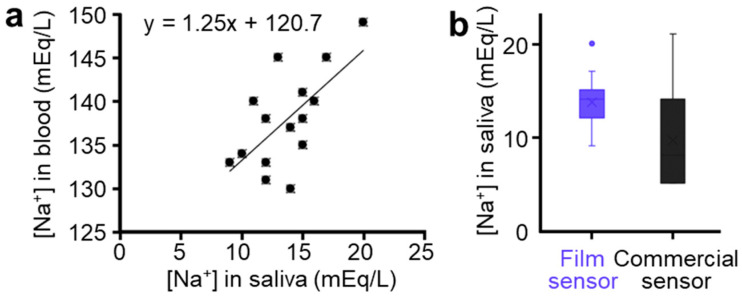
(**a**) Correlation of sodium ion levels measured from blood and saliva. (**b**) Correlation of sodium in levels measured from our sensor and commercial sensor.

This study shows the results of measured ion levels from sixteen infants regarding the correlation between blood and saliva. The ion levels measured using unstimulated whole saliva with our sensor are compared with blood measured within an hour. To predict the correlation between saliva and blood ion levels, the Spearman correlation analysis is performed for those subjects, considering a correlation significance, if a coefficient >0.50. The summarized result in Figure 4a captures a positive correlation in blood-to-saliva ion levels with a correlation coefficient of 0.563 (*p* = 0.023). In addition, this study verifies that ion levels measured by the flexible film sensor are comparable to a commercial ion sensor. Both values from multiple subjects showed the general sodium levels in saliva, as shown in Figure 4b. The measured ion levels resulted in the normal range of salivary sodium of 13.69 ± 2.75 and 9.58 ± 5.23 mEq/L via our film and commercial sensors, respectively. The correlation in film sensor-to-commercial sensor shows a significant correlation coefficient of 0.594 (*p* = 0.042). Table 1 compares the non-invasive sensing capability of the salivary sodium detection system between our flexible SS-ISE and published systems. Note that the detection of the salivary ions relied on the bulky systems despite its diagnosis possibility, which was confirmed by the positive blood-to-saliva correlation [12,14,15,16]. Overall, the correlation and validation results confirm that our small film sensors can be used in clinical trials while reporting the correlation in blood-to-saliva ion levels for the first time from an SS-ISE system.

## 4. Conclusions

In this study, we report that salivary sodium levels, measured by a flexible film sensor system, are comparable to those from blood, which was validated by a commercial device. The flexible system, including electrochemical sensors and a Bluetooth-enabled wireless circuit, has an exceptionally small form factor, showing an excellent possibility for non-invasive, portable disease diagnosis. This study demonstrates a reliable performance of the new sensors (RE and ISE) via a structural characterization and a direct comparison with a commercial sensor. In addition, the high correlation of measured sodium ions between saliva and blood shows the potential of the film sensor system for a non-invasive clinical study with a large patient population. The presented potentiometric sensor system using saliva, where any volumetric change in sample solutions highly affects the results, could enable the rapid detection of collected samples from the general population. The use of the low-profile, flexible sensor system will provide more comfort in terms of easy deployment on any surface of interest as well as seamless wearability. Future work will focus on further miniaturization of the wireless sensor system and integration of soft microfluidic channels and low-power circuits for continuous, real-time detection of sodium levels for pediatric health monitoring.

## Figures and Tables

**Table 1:** Design comparison of non-invasive, salivary sodium detection system between this work and prior reports.

Reference	Design			Non-Invasive Sodium Sensing Performance	
	Sensor	Device Size	Wireless Monitoring	[Na^+^] (mEq/L) ^(1)^	Correlation to Blood
This work	Flexible SS-ISE	4 × 5.5 cm^2^	Yes	13.69 ± 2.75	Linear
[18]	Flexible SS-ISE ^(2)^	3 × 2 cm^2^	Yes	6.5–11.8	-
[37]	SS-ISE	Table-top	No	0.8 ± 0.1	-
[38]	SS-ISE	Table-top	No	1.97–10.32	-
[12]	ISE	Table-top	No	10–13	-
[13]	ECL ^(3)^	Table-top	No	6–35	-
[16]	Photometer	Table-top	No	11.5–217.3	Linear
[14]	Photodetector	Table-top	No	10–30	Linear
[15]	Radiometer	Table-top	No	12.26 ± 4.31	Linear
[12]	Radiometer	Table-top	No	12.87 ± 5.85	Linear

^(1)^ Standardizing the sampling methods is required. ^(2)^ Our previous work. ^(3)^ Electrochemiluminescence.

## Data Availability

Deidentified participant data will be available from the corresponding author on formal request. The data will not be publicly available due to ethical considerations.

## Supplementary Materials

The following are available online at https://www.mdpi.com/1424-8220/21/5/1642/s1. Figure S1: Fabrication process; Figure S2: Illustration of a wireless circuit; Figure S3: Sensor repeatability and detection range; Table S1: List of circuit components.
