# Supplementary material for: Development of Flexible Ion-Selective Electrodes for Saliva Sodium Detection
Source: Sensors (Basel). 2021 Feb 26;21(5):1642. doi: 10.3390/s21051642 (PMC7956447; doi:10.3390/s21051642)
Supplement: Supplementary file 1 [file sensors-21-01642-s001.pdf]

**Supporting Information**

# Development of Flexible Ion-Selective Electrodes for Saliva Sodium Detection

**Hyo-Ryoung Lim [1,6], Soon Min Lee [2,6], Musa Mahmood [1], Shinjae Kwon [1], Yun-Soung Kim [1], Yongkuk Lee [3] and Woon-Hong Yeo [1,4,5*]**

1 George W. Woodruff School of Mechanical Engineering, Center for Human-Centric Interfaces and Engineering, Institute for Electronics and Nanotechnology, Georgia Institute of Technology, Atlanta, GA 30332, USA; hlim308@gatech.edu (H.-R.L.); musamahmood@gatech.edu (M.M.); skwon64@gatech.edu (S.K.)

2 Department of Pediatrics, Gangnam Severance Hospital, Yonsei University College of Medicine, Seoul 06273, Korea; smlee@yuhs.ac

3 Department of Biomedical Engineering, Wichita State University, Wichita, KS 67260, USA; yongkuk.lee@wichita.edu

4 Wallace H. Coulter Department of Biomedical Engineering, Georgia Institute of Technology and Emory University School of Medicine, Atlanta, GA 30322, USA

5 Parker H. Petit Institute for Bioengineering and Biosciences, Institute for Materials, Neural Engineering Center, Institute for Robotics and Intelligent Machines, Georgia Institute of Technology, Atlanta, GA 30332, USA

6 These authors contributed equally to this work

* Correspondence: whyeo@gatech.edu; Tel.: +1-404-385-5710; Fax: +1-404-894-1658

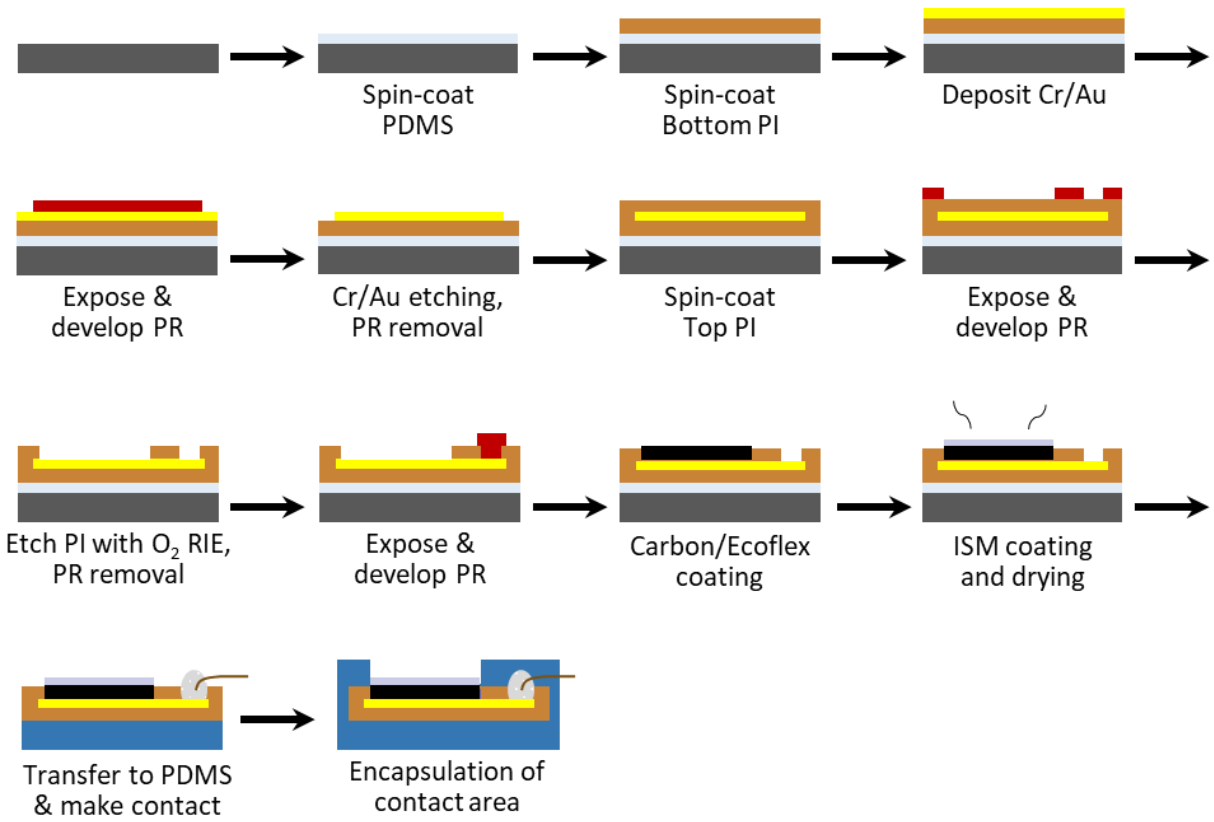


**Figure S1. Fabrication processes for a flexible film ISE.**

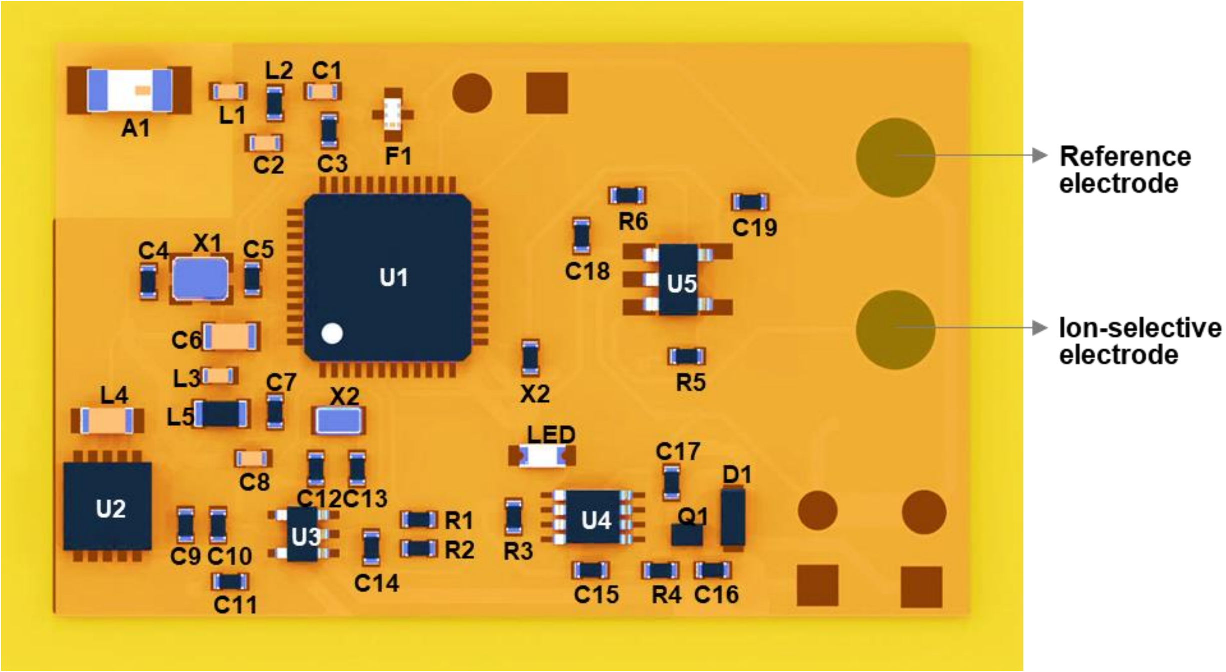


**Figure S2. Illustration of wireless, low-power sensor system components with a detailed list of the surface mount chip components in Table S1.**

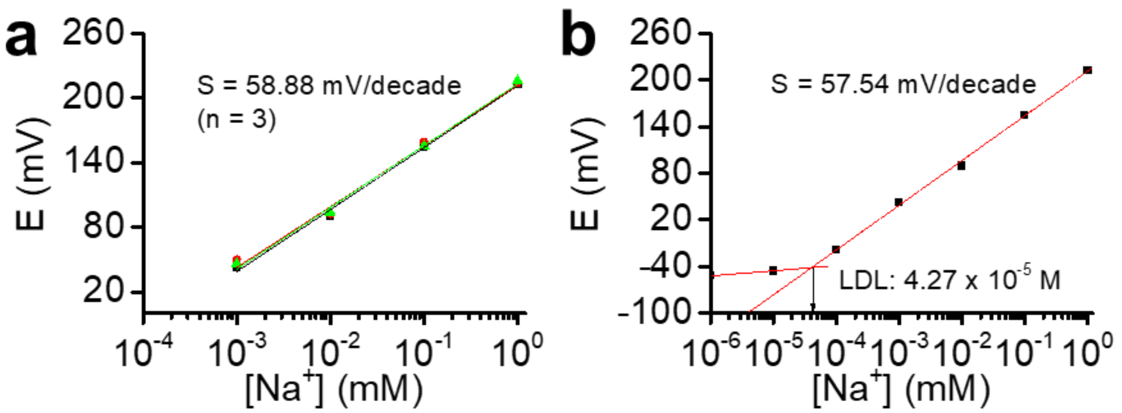


**Figure S3. (a) Repeatability, (b) detection range, and low detection limit (LDL) of a flexible SS-ISE.**

**Table S1. List of surface mount components used in the wireless, low-power sensor system.**

| Component | Description | Value | Part number |
|---|---|---|---|
| U1 | Bluetooth PSoC | N/A | NRF52832-QFAA-R |
| U2 | 3.3 voltage regulator | N/A | TPS63001 |
| U3 | Current limit active-low load switch | N/A | TPS22941 |
| U4 | Battery recharge | N/A | MCP73831 |
| U5 | Single channel Op-amp IC | N/A | AD8603AUJZREEL7 |
| F1 | 2.45 GHz low pass filter | N/A | 2450FM07A0029 |
| A1 | 2.45 GHz RF chip antenna | N/A | 2450AT18A100 |
| D1 | Schottky Diode | N/A | 641-1285-1-ND |
| Q1 | P-MOSFET | N/A | DMP21D5UFB4-7BDICT-ND |
| X1 | 32 MHz crystal | N/A | ECS-320-8-37CKM |
| X2 | 32.768 kHz crystal | N/A | ECS-327-9-12-TR |
| L1 | 0402 inductor | 3.9 nH | N/A |
| L2 | 0402 inductor | 2.7 nH | N/A |
| L3 | 0402 inductor | 15 nH | N/A |
| L4 | 0603 inductor | 2.2 μH | N/A |
| L5 | 0603 inductor | 10 μH | N/A |
| C1 | 0402 ceramic capacitor | 1.0 pF | N/A |
| C2, C8 | 0402 ceramic capacitor | 100 nF | N/A |
| C3 | 0402 ceramic capacitor | 100 pF | N/A |
| C4, C5, C12, C13 | 0402 ceramic capacitor | 12 pF | N/A |
| C6 | 0603 ceramic capacitor | 1.0 μF | N/A |
| C7, C15, C17 | 0402 ceramic capacitor | 4.7 μF | N/A |
| C9 | 0402 ceramic capacitor | 22 μF | N/A |
| C10, C11, C14, C18 | 0402 ceramic capacitor | 10 μF | N/A |
| C16 | 0402 ceramic capacitor | 1.0 μF | N/A |
| C19 | 0402 ceramic capacitor | 10 nF | N/A |
| R1, R2 | 0402 resistor | 1 MΩ | N/A |
| R3 | 0402 resistor | 2 kΩ | N/A |
| R4 | 0402 resistor | 100 kΩ | N/A |
| R5, R6 | 0402 resistor | 10 kΩ | N/A |
| Battery | Lithium-ion polymer | | |
